# Supplementary material for: Assessing the Occurrence and Influence of Cancer Chemotherapy-Related Pharmacogenetic Alleles in the Chilean Population
Source: Pharmaceutics. 2024 Apr 19;16(4):561. doi: 10.3390/pharmaceutics16040561 (PMC11054647; doi:10.3390/pharmaceutics16040561)
Supplement: Supplementary file 1 [file pharmaceutics-16-00561-s001.zip › pharmaceutics-2932680-supplementary.pdf]

Supplementary Materials

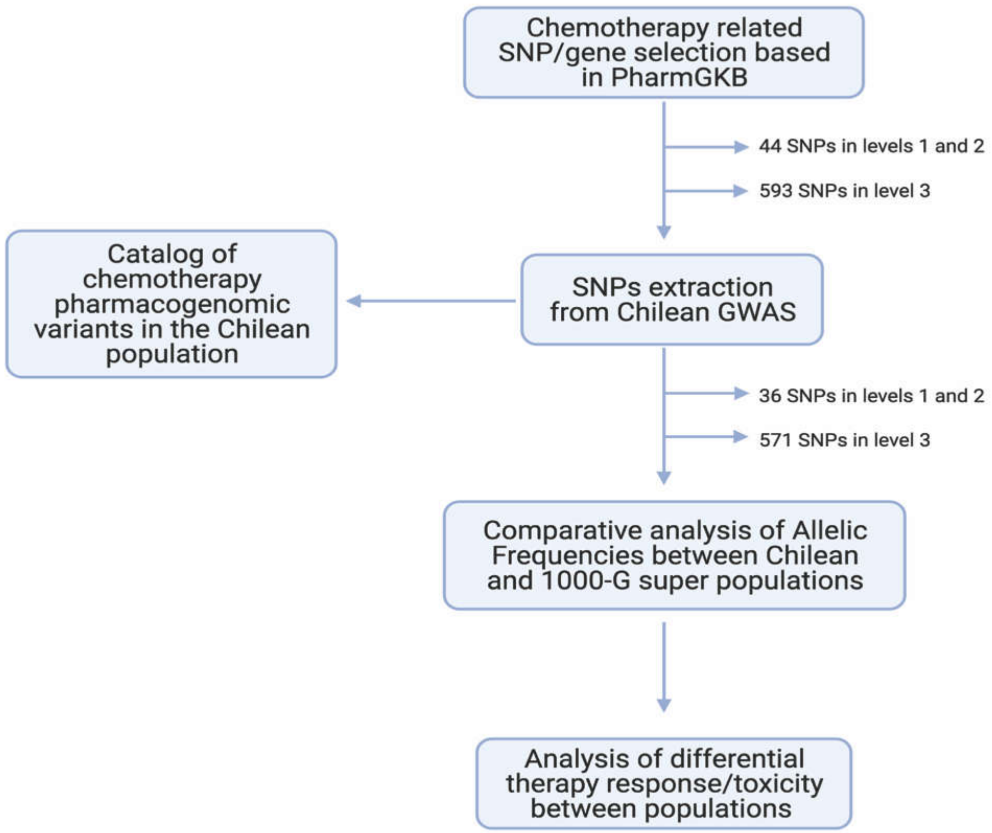

Figure S1. General design of the study. PharmGKB database was used for the SNP selection related to response/toxicity of cancer therapy. Then, allelic frequencies of selected SNPs were extracted from Chilean GWAS of 1095 non-cancerous subjects, and significant differences were established with respect to the 5 super populations of 1000G-project. Finally, a discussion with relevant literature was performed.

Table S1. Minor allele frequencies of pharmacogene SNPs level of evidence 1 and 2.

Gene	SNP	MA	CHI	AMR	EUR	EAS	SAS	AFR
ABCB1	rs1045642	A	0.394	0.428	0.514	0.399	0.576	0.151
ACYP2	rs1872328	A	0.023	0.043	0.038	0.012	0.017	0.198
C8orf34	rs1517114	C	0.400	0.338	0.356	0.122	0.309	0.424
CBR3	rs1056892	A	0.278	0.260	0.355	0.400	0.530	0.509
CEP72	rs924607	T	0.399	0.319	0.409	0.305	0.376	0.077
CYP2A6	rs1801272	T	0.018	0.007	0.034	0.000	0.006	0.001
CYP2A6	rs28399433	C	0.092	0.099	0.072	0.235	0.153	0.083
CYP2A6	rs28399454	T	0.001	0.006	0.000	0.001	0.000	0.116
CYP2D6	rs28371725	T	0.050	ND	ND	ND	ND	ND
CYP2D6	rs35742686	C	0.007	ND	ND	ND	ND	ND
CYP2D6	rs3892097	T	0.140	ND	ND	ND	ND	ND
CYP2D6	rs5030655	C	0.005	ND	ND	ND	ND	ND
CYP2D6	rs1065852	A	0.153	ND	ND	ND	ND	ND
DPYD	rs75017182	C	0.005	0.006	0.024	0.000	0.019	0.001
DPYD	rs67376798	A	0.003	0.003	0.007	0.000	0.001	0.001
ERCC1	rs11615	A	0.401	0.388	0.619	0.262	0.460	0.036
ERCC1	rs3212986	A	0.390	0.353	0.251	0.300	0.299	0.291
FCGR2A	rs1801274	G	0.406	0.451	0.513	0.278	0.419	0.527
FCGR3A	rs396991	C	0.323	0.267	0.360	0.373	0.331	0.326
GSTP1	rs1695	G	0.426	0.474	0.330	0.180	0.294	0.484
MTHFR	rs1801133	A	0.426	ND	ND	ND	ND	ND
NQO1	rs1800566	A	0.407	0.332	0.211	0.419	0.358	0.176
NT5C2	rs11598702	C	0.314	0.309	0.349	0.233	0.236	0.186
NUDT15	rs116855232	T	0.062	0.045	0.003	0.094	0.070	0.001
NUDT15	rs147390019	A	0.004	0.009	0.000	0.001	0.000	0.000
NUDT15	rs869320766	AGGAGTC	0.061	ND	ND	ND	ND	ND
SEMA3C	rs7779029	C	0.061	0.082	0.045	0.152	0.107	0.360
SLC28A3	rs885004	A	0.182	0.201	0.129	0.159	0.102	0.100

SLC28A3	rs7853758	A	0.191	0.233	0.142	0.155	0.112	0.339
SLCO1B1	rs11045879	C	0.135	0.174	0.193	0.450	0.081	0.187
TPMT	rs1800462	G	0.001	0.006	0.007	0.000	0.000	0.001
TPMT	rs1142345	C	0.037	0.057	0.029	0.021	0.017	0.066
TPMT	rs1800460	T	0.031	0.040	0.028	0.000	0.004	0.003
UGT1A1	rs4148323	A	0.025	0.011	0.007	0.136	0.017	0.001
UGT1A9	rs3832043	A	0.448	ND	ND	ND	ND	ND
XRCC1	rs25487	T	0.243	0.313	0.361	0.236	0.343	0.110

MA = minor allele, CHI = Chilean, AMR = Admixed Americans, EUR = Europeans, EAS = East Asians, SAS = South Asians, and AFR = Africans, ND = No data

Table S2. Significant SNPs showing positive association with Mapuche-Huilliche ancestry.

Gene	SNP	MA	Test	Beta	SE	L95	U95	Stat	*P*
ABCB1	rs2235047	C	ADD	0.006306	0.001775	0.002827	0.009785	3.553	0.0003974
NUDT15	rs116855232	T	ADD	0.01086	0.00307	0.004847	0.01688	3.539	0.0004187
NAT2	rs1799931	A	ADD	0.006863	0.002291	0.002373	0.01135	2.996	0.002799
RAD52	rs11226	A	ADD	0.00446	0.001493	0.001533	0.007387	2.986	0.002885
NT5C3A	rs3750117	A	ADD	0.004406	0.001486	0.001494	0.007318	2.966	0.003084
RXRA	rs2234753	G	ADD	0.004488	0.001517	0.001516	0.007461	2.96	0.003148
DROSHA	rs639174	T	ADD	0.004147	0.001489	0.001229	0.007064	2.786	0.005437
KCNQ5	rs9351963	C	ADD	0.004425	0.001616	0.001258	0.007593	2.738	0.006282
TP53	rs4968187	T	ADD	0.004785	0.00176	0.001336	0.008235	2.719	0.006654
NUDT15	rs869320766	AGGAGTC	ADD	0.008037	0.003133	0.001898	0.01418	2.566	0.01043
FKBP15	rs4978536	G	ADD	0.003858	0.001548	0.0008248	0.006891	2.493	0.01282
CYP1A1	rs1048943	C	ADD	0.003803	0.001584	0.0006978	0.006908	2.4	0.01654
CCAT2	rs6983267	T	ADD	0.003469	0.001481	0.0005669	0.006371	2.343	0.01932
SLCO1B1	rs10841753	C	ADD	0.003701	0.001651	0.0004648	0.006937	2.241	0.0252
HOTAIR	rs7958904	C	ADD	0.003177	0.001488	0.0002615	0.006093	2.136	0.03293

BCL2L11	rs724710	T	ADD	0.003154	0.001492	0.0002297	0.006079	2.114	0.03475
NQO1	rs1800566	A	ADD	0.003037	0.00148	0.0001372	0.005937	2.053	0.04034
EPHX1	rs1051740	C	ADD	0.003012	0.001509	5.44E-05	0.00597	1.996	0.04618

MA = minor allele, Test = linear additive test, Beta = effect size, SE = standard error of beta, L95 = 95% confidence interval (lower bound), U95 = 95% confidence interval (upper bound), Stat = regression statistic.

Table S3. Significant SNPs showing positive association with European ancestry.

Gene	SNP	MA	Test	Beta	SE	L95	U95	Stat	*P*
GSTA1	rs3957357	A	ADD	0.007623	0.001642	0.004405	0.01084	4.642	3.87E-06
PARD3B	rs17626122	T	ADD	0.005688	0.001507	0.002734	0.008642	3.774	0.0001693
NALCN	rs7992226	G	ADD	0.006763	0.001861	0.003116	0.01041	3.634	0.0002918
FOXO1; MIR320D1	rs144991623	T	ADD	0.05285	0.01525	0.02296	0.08273	3.466	0.0005492
OTOS	rs2291767	C	ADD	0.01767	0.005487	0.006913	0.02842	3.22	0.001321
GSTP1	rs1138272	T	ADD	0.01308	0.004149	0.004945	0.02121	3.152	0.001667
UBE2I	rs9597	G	ADD	0.01575	0.005144	0.005669	0.02583	3.062	0.002252
KDR	rs2071559	G	ADD	0.004373	0.0015	0.001433	0.007314	2.915	0.003633
ABCC1	rs17501331	G	ADD	0.008602	0.003033	0.002657	0.01455	2.836	0.004655
CYBA	rs4673	A	ADD	0.004563	0.00168	0.001271	0.007855	2.716	0.006706
CYP1A2	rs2472304	A	ADD	0.004139	0.001528	0.001144	0.007135	2.708	0.00687
NOS3	rs1799983	T	ADD	0.004863	0.001796	0.001342	0.008384	2.707	0.006899
XPC	rs2228001	G	ADD	0.004206	0.001604	0.001062	0.007351	2.622	0.008864
IRX5; IRX6	rs9936750	C	ADD	0.006143	0.00237	0.001497	0.01079	2.591	0.009685
MUTYH	rs3219484	T	ADD	0.01224	0.004814	0.002803	0.02168	2.542	0.01115
FKBP15	rs4979223	A	ADD	0.003689	0.001493	0.0007634	0.006615	2.471	0.01361
LOC102723649; LINC02539	rs6920220	A	ADD	0.005496	0.002257	0.001072	0.009919	2.435	0.01505
WWOX; MAF	rs4888024	A	ADD	0.003505	0.001447	0.0006698	0.00634	2.423	0.01556
PIN1	rs2233678	C	ADD	0.00625	0.002605	0.001144	0.01136	2.399	0.0166
FLT4	rs307821	A	ADD	0.007445	0.003108	0.001353	0.01354	2.395	0.01677

PERP; ARFGEF3	rs117101815	T	ADD	0.01013	0.004239	0.001822	0.01844	2.39	0.01703
PERP; ARFGEF3	rs9402944	T	ADD	0.01013	0.004239	0.001822	0.01844	2.39	0.01703
KDR	rs2239702	T	ADD	0.004043	0.00172	0.0006718	0.007414	2.351	0.01892
PERP; ARFGEF3	rs78428806	A	ADD	0.01	0.004266	0.001642	0.01836	2.345	0.01921
COMT	rs4646316	T	ADD	0.00445	0.001907	0.0007122	0.008188	2.333	0.01981
ABCC2	rs717620	T	ADD	0.004791	0.002068	0.0007383	0.008844	2.317	0.02069
ABCC2	rs2273697	A	ADD	0.004736	0.002088	0.0006437	0.008828	2.268	0.02351
XYLT2	rs6504649	G	ADD	0.003653	0.00164	0.0004389	0.006867	2.228	0.02611
PACSIN2	rs2413739	T	ADD	0.003455	0.001609	0.0003011	0.006608	2.147	0.03201
MTRR	rs1801394	G	ADD	0.003335	0.001559	0.0002791	0.006391	2.139	0.03266
SLC22A4	rs1050152	T	ADD	0.00363	0.001724	0.0002522	0.007009	2.106	0.03541
EGFR	rs712829	T	ADD	0.003538	0.001698	0.000211	0.006865	2.084	0.03737
NFKBIA	rs2233409	A	ADD	0.004541	0.002188	0.0002525	0.008829	2.075	0.03819
XRCC3	rs861539	A	ADD	0.003671	0.001771	0.000199	0.007143	2.072	0.03847
SLC31A1	rs10981694	G	ADD	0.004685	0.002262	0.0002504	0.009119	2.071	0.03863
DCK	rs80143932	G	ADD	0.005395	0.002644	0.0002118	0.01058	2.04	0.04159
DCK	rs2306744	T	ADD	0.005395	0.002644	0.0002118	0.01058	2.04	0.04159
E2F7	rs310786	C	ADD	0.003483	0.001709	0.0001331	0.006832	2.038	0.04181
LINC02247;RXRA	rs62576288	A	ADD	0.006378	0.003131	0.000242	0.01251	2.037	0.04187
NCF4-AS1	rs1883112	G	ADD	0.00299	0.001512	2.68E-05	0.005954	1.978	0.04821
SLC31A1	rs7851395	A	ADD	0.002974	0.001507	1.95E-05	0.005928	1.973	0.04876

MA = minor allele, Test = linear additive test, Beta = effect size, SE = standard error of beta, L95 = 95% confidence interval (lower bound), U95 = 95% confidence interval (upper bound), Stat = regression statistic.

Table S4: Significant pharmacogenes SNPs related to European ancestry in the Chilean cohort.

Gene	SNP	*P**	MA	LoE	Drug	PharmGKB annotations**
ABCC1	rs17501331	0.00466	G	3	Irinotecan	Patients with the AA genotype and colorectal neoplasms may have increased severity of neutropenia compared to patients with the AG and GG genotypes when taking irinotecan.
ABCC2	rs717620	0.02069	T	3	Fluorouracil / Leucovorin / Oxaplatin / Cisplatin/ Doxorubicin / Methotrexate / Sorafenib	Patients with TT genotype and colorectal cancer may have increased severity of neurotoxicity syndromes when treated with FOLFOX (fluorouracil, leucovorin, oxaliplatin) as compared to patients with CC or CT genotype.<br>Patients with the CC genotype and colon cancer may have an increased risk of thrombocytopenia when treated with FOLFOX as compared to patients with CT or TT genotype.<br>Patients with osteosarcoma and CT or TT genotype may have a decreased response to treatment with cisplatin, doxorubicin and methotrexate as compared to patients with the CC genotype.<br>Patients with the CC genotype may have increased risk of skin rash when treated with sorafenib in people with Carcinoma, Renal Cell as compared to patients with genotype CT or TT.
ABCC2	rs2273697	0.02351	A	3	Cisplatin / Imatinib / Cyclophosphamide / Doxorubicin / Fluorouracil	Patients with mesothelioma and the GG genotype may have worse overall and progression-free survival when treated with cisplatin as compared to patients with the AA or AG genotypes.<br>Patients with the GG genotype and gastrointestinal stromal tumors may have decreased progression-free survival times when treated with imatinib as compared to patients with the AA or AG genotype.<br>Patients with GG genotype and breast cancer may have an increased risk of anemia when treated with cyclophosphamide, doxorubicin and fluorouracil (FAC) as compared to patients with AA or AG genotype.
COMT	rs4646316	0.01981	T	3	Cisplatin	The TT genotype may be associated with increased likelihood of nephrotoxicity when treated with cisplatin as compared to the CT or TT genotype.
CYBA	rs4673	0.00671	A	3	Doxorubicin / Idarubicin / Cisplatin	Cancer patients with the AA or AG genotypes who are treated with doxorubicin may have an increased risk for cardiotoxicity as compared to patients with the GG genotype.<br>Cancer patients with the AA genotype may have a shorter overall and event-free survival time as compared to patients with the AG or GG genotype when treated with anthracyclines.<br>Patients with osteosarcoma and GG genotype may be at an increased risk of experiencing side effects when treated with cisplatin and doxorubicin as compared to patients with the AA or AG genotypes.
CYP1A2	rs2472304	0.00687	A	3	Erlotinib	Patients with GG genotype may have decreased concentrations of erlotinib as compared to patients with AA or AG genotype.
DCK	rs80143932	0.04159	G	3	Cytarabine / Idarubicin	Patients with acute myeloid leukemia and the GG genotype may have an increased response to cytarabine and idarubicin as compared to patients with the CC genotype. Other clinical and genetic factors may also influence response in patients administered cytarabine and idarubicin.
DCK	rs2306744	0.04159	T	3	Cytarabine / Idarubicin	Patients with acute myeloid leukemia and the CC genotype may have a decreased response to cytarabine and idarubicin as compared to patients with the TT genotype. Other clinical and genetic factors may also influence response in patients administered cytarabine and idarubicin.
E2F7	rs310786	0.04181	C	3	Tamoxifen	Women with the CC genotype and breast cancer may have increased lumbar bone loss when treated with tamoxifen as compared to women with the CT or TT genotype. Other genetic and clinical factors may also influence lumbar bone loss in women taking tamoxifen.

EGFR	rs712829	0.03737	T	3	Erlotinib / Cetuximab / Irinotecan	Patients with the GG genotype may be less sensitive to treatment with erlotinib compared to patients with the GT or TT genotype. Patients with GG genotype and colorectal cancer may have a shorter overall survival and progression-free survival time when receiving anti-EGFR plus irinotecan, as compared to patients with GT or TT genotype.
FLT4	rs307821	0.01677	A	3	Sunitinib	Patients with AC genotype and Renal Cell Carcinoma who are treated with sunitinib may have reduced progression-free survival as compared to patients with the CC genotype.
FOXO1	rs144991623	0.00055	T	3	Cyclophosphamide / Epirubicin / Fluorouracil	Patients with breast cancer and TT genotype may have an increased risk of developing neutropenia when treated with cyclophosphamide, epirubicin and fluorouracil as compared to patients with CC or CT genotypes.
GSTA1	rs3957357	$3.87E^{-06}$	A	3	Cisplatin / Cyclophosphamide / Doxorubicin / Rituximab	Patients with the AA or AG genotypes may have increased risk for anemia when treated with cisplatin and cyclophosphamide as compared to patients with the GG genotype. Patients with the GG genotype and diffuse large B-cell lymphoma may have a shorter event-free survival time when treated with the R-CHOP chemotherapy regimen as compared to patients with the AA or AG genotypes. Patients with the AG or GG genotypes and soft tissue sarcoma may have a shorter progression-free survival time when treated with doxorubicin as compared to patients with the AA genotype.
GSTP1	rs1138272	0.00167	T	3	Cisplatin	Patients with the CT or TT genotypes and cancer may have shorter survival times when treated with cisplatin as compared to patients with the CC genotype.
IRX5	rs9936750	0.00969	C	3	Capecitabine	Patients with the CC or CT genotype who are treated with capecitabine may have an increased risk for capecitabine-induced toxicity as compared to patients with the TT genotype.
KDR	rs2239702	0.00363	T	3	Sorafenib	Patients with the CT or TT genotypes may have unfavorable progression-free survival and overall survival when treated with sorafenib in people with Carcinoma, Renal Cell as compared to patients with genotype CC.
KDR	rs2071559	0.01892	G	3	Sorafenib	Patients with AA or AG genotypes may have decreased overall survival and progression-free survival when treated with sorafenib in people with Carcinoma, Renal Cell or hepatocellular carcinoma as compared to patients with the GG
MAF	rs4888024	0.01556	A	3	Methotrexate	Patients with the AA genotype and childhood acute lymphoblastic leukemia who are treated with methotrexate may have a decreased, but not absent, risk of end-of-induction minimal residual disease (MRD) as compared to patients with the AG or GG genotype. Other genetic and clinical factors may also influence a patient's risk for MRD.
MTRR	rs1801394	0.03266	G	3	Sevoflurane	Patients with the rs1801394 GG genotype who are treated with sevoflurane may have decreased mean arterial pressure as compared to patients with the AA genotype. Other genetic and clinical factors may also influence mean arterial pressure.
MUTYH	rs3219484	0.01115	T	3	Cisplatin / Cyclophosphamide	Patients with CC genotype and ovarian cancer who are treated with cisplatin and cyclophosphamide may have an increased risk of grade 3-4 neutropenia as compared to patients with CT genotype.
NALCN	rs7992226	0.00029	G	3	Methotrexate	Patients with the GG genotype and childhood acute lymphoblastic leukemia (ALL) may have increased exposure to methotrexate and lower likelihood of minimal residual disease as compared to patients with the AA genotype. Other genetic and clinical factors may also influence clearance of methotrexate.

NCF4	rs1883112	0.04821	G	3	Doxorubicin / Idarubicin	Cancer patients with AA genotype who are treated with doxorubicin or idarubicin may have an increased risk for cardiotoxicity as compared to patients with AG and GG genotype.
NFKBIA	rs2233409	0.03819	A	3	Gefitinib	Patients with non-small cell lung cancer and the AA genotype may be at an increased risk of developing diarrhea when treated with gefitinib as compared to patients with the AG or GG genotypes.
NOS3	rs1799983	0.00690	T	3	Cyclophosphamide / Doxorubicin / Fluorouracil / Sorafenib	Patients with TT genotype may have shorter disease-free survival when treated with cyclophosphamide-based regimens, as compared to patients with the GG or GT genotype. Hepatocellular Carcinoma patients with GG genotype may have decreased progression-free survival and decreased overall survival when treated with sorafenib as compared to patients with genotype GT or TT.
OTOS	rs2291767	0.001321	C	3	Cisplatin	Cancer patients with the TT genotype may have an increased risk of ototoxicity when treated with cisplatin as compared to patients with the CC or CT genotypes.
PACSIN2	rs2413739	0.03201	T	3	Mercaptopurine	Patients with Precursor Cell Lymphoblastic Leukemia-Lymphoma and TT genotype may have increased risk of adverse events when treated with mercaptopurine as compared to patients with the CC or CT genotypes.
PARD3B	rs17626122	0.00017	T	3	Fluorouracil / Oxiplatin	Patients with the CT or TT genotypes may have increased likelihood of Drug Toxicity when treated with fluorouracil and oxaliplatin in people with Colorectal Neoplasms as compared to patients with genotype CC.
PERP	rs117101815	0.01703	T	3	Cyclophosphamide / Epirubicin / Fluorouracil	Patients with breast cancer and GG or GT genotypes may have an increased risk of developing neutropenia when treated with cyclophosphamide, epirubicin and fluorouracil as compared to patients TT genotype.
PERP	rs9402944	0.01703	T	3	Cyclophosphamide / Epirubicin / Fluorouracil	Patients with breast cancer and GG or GT genotypes may have an increased risk of developing neutropenia when treated with cyclophosphamide, epirubicin and fluorouracil as compared to patients TT genotype.
PERP	rs78428806	0.01921	A	3	Cyclophosphamide / Epirubicin / Fluorouracil	Patients with breast cancer and GG genotype may have an increased risk of developing neutropenia when treated with cyclophosphamide, epirubicin and fluorouracil as compared to patients with AA or AG genotypes.
PIN1	rs2233678	0.0166	C	3	Irinotecan / Oxaliplatin	Patients with the GG genotype and colorectal cancer may have decreased survival times when treated with irinotecan-based treatments as compared to patients with the CC or CG genotypes. Patients with the CC or CG genotypes and colorectal cancer may have decreased survival times when treated with oxaliplatin-based treatments as compared to patients with the GG genotype.
RXRA	rs62576288	0.04187	A	3	Docetaxel	Patients with nasopharyngeal cancer and the GG genotype who are treated with docetaxel may have more severe anemia as compared to the AA genotype.
SLC22A4	rs1050152	0.03541	T	3	Imatinib	Patients with the TT genotype and cancer may have a poorer response to treatment with imatinib as compared to patients with the CC or CT genotype.
SLC31A1	rs4979223	0.01361	A	3	Platinum Compounds	Patients with the AC genotype and non-small cell lung cancer who are treated with platinum compounds may have increased severity of thrombocytopenia, and decreased likelihood of overall survival as compared to patients with AA or CC genotypes.
SLC31A1	rs10981694	0.03863	G	3	Cisplatin	Patients with the GG or GT genotypes and NSCLC who are treated with cisplatin may have an increased risk of severe ototoxicity as compared to patients with the TT genotype.

SLC31A1	rs7851395	0.04876	A	3	Carboplatin / Cisplatin	Patients with the AG or GG genotypes may have decreased overall survival when treated with carboplatin or cisplatin in people with Non-Small-Cell Lung Carcinoma as compared to patients with genotypes AA.
TNFAIP3	rs6920220	0.01505	A	3	Methotrexate	Patients with the AA genotype may have increased likelihood of discontinuation of methotrexate in people with Arthritis as compared to patients with genotype GG. Other genetic and clinical factors may also influence the response to methotrexate.
UBE2I	rs9597	0.00225	G	3	Cisplatin / Irinotecan	Patients with the CC genotype may have a decreased response to cisplatin and irinotecan as compared to patients with the CG or GG genotypes.
XPC	rs2228001	0.00886	G	3	Cisplatin	Patients with bladder cancer and the GG or GT genotypes may be at an increased risk of developing neutropenia when treated with cisplatin as compared to patients with the TT genotype.<br>Patients with bladder cancer and the GG or GT genotypes may be at an increased risk of experiencing drug toxicity when treated with cisplatin as compared to patients with the TT genotype.
XRCC3	rs861539	0.03847	A	3	Fluorouracil / Irinotecan / Leucovorin / Platinum Compounds	Patients with GG genotype may have decreased progression free survival in people with colorectal cancer when treated with fluorouracil, irinotecan and leucovorin as compared to patients with AA or AG genotype.<br>Patients with the GG genotype and non-small cell lung cancer may have a worse response when treated with platinum compounds as compared to patients with the AA or AG genotypes.
XYLT2	rs6504649	0.02611	G	3	Carboplatin / Gemcitabine	Patients with non-small cell lung cancer and CG or GG genotype may experience an increased severity of thrombocytopenia when treated with carboplatin and gemcitabine as compared to patients with CC genotype.

MA: minor allele; LoE: level of evidence; SNP: single nucleotide polymorphism. P*; P-value for the association between the pharmacogene SNPs genotypes and the European ancestry.
